# Rapamycin treatment early in life reprograms aging: hyperfunction theory and clinical practice

**DOI:** 10.18632/aging.204354

**Published:** 2022-10-24

**Authors:** Mikhail V. Blagosklonny

**Affiliations:** 1 Roswell Park Comprehensive Cancer Center, Buffalo, NY 14263, USA

**Keywords:** senescence, gerostatics, geroscience, sirolimus, healthspan

## Abstract

Making provocative headlines, three outstanding publications demonstrated that early-life treatment with rapamycin, including treatments during developmental growth, extends lifespan in animals, confirming predictions of hyperfunction theory, which views aging as a quasi-program (an unintended continuation of developmental growth) driven in part by mTOR. Despite their high theoretical importance, clinical applications of two of these studies in mice, *Drosophila* and *Daphnia* cannot be implemented in humans because that would require growth retardation started at birth. A third study demonstrated that a transient (around 20% of total lifespan in *Drosophila*) treatment with rapamycin early in *Drosophila* adult life is as effective as lifelong treatment, whereas a late-life treatment is not effective. However, previous studies in mice demonstrated that a transient late-life treatment is highly effective. Based on hyperfunction theory, this article attempts to reconcile conflicting results and suggests the optimal treatment strategy to extend human lifespan.

## INTRODUCTION

### “Brief” treatment in flies and mice

In September 2022, provocative headlines declared: Brief exposure to rapamycin has the same anti-aging effects as lifelong treatment, shows study in fruit flies and mice.

Other news announced: Less is more: early short-course rapamycin effective in sustained anti-aging effects.

“We have found a way to circumvent the need for chronic, long-term rapamycin intake, so it could be more practical to apply in humans,” says Dr. Yu-Xuan Lu, a co-author of one of the papers.

This seems exciting on the surface. However, according to these well-executed studies in mice and flies, rapamycin treatment should be started at birth (or shortly after birth) to be most effective [1–3]. So, it’s definitely too late for anyone capable of reading this article. Noteworthy, the “brief” treatment is not brief, it is an equivalent to 3–20 years of human life, as we will discuss. Furthermore, started at birth, rapamycin treatment severely inhibited developmental growth [2, 3].

What is remarkable though is that lifelong treatment with rapamycin was safe (no increase in mortality) and effective (an increase in longevity) in mice [2] and *Drosophila* [1, 2]. The results of these studies agree with the predictions of hyperfunction theory of mTOR-driven quasi-program of aging. According to hyperfunction theory, the quasi-program of aging is a purposeless, unintended continuation of the developmental growth program that was not switched off upon its completion [4–8]. As mentioned in 2006, “Once development is completed, a program for development is not switched off, thus becoming a quasi-program for aging. This hyper-functional quasi- program is manifested as diseases of aging, leading to damage and secondary decline.” [4]. By slowing developmental growth, the quasi-program of aging can be re-programmed for a slower pace. (Note: quasi-program or pseudo-program is an undirected continuation of a program for something else, e.g., developmental growth [4]).

Rapamycin treatment (for a duration of 30 days and 15 days) started at day 3 after eclosion (the emergence of an adult fly from its pupal case) and increased lifespan as much as lifelong treatment did in *Drosophila* [1]. (Note: Given the short lifespan of *Drosophila*, this can be compared to treatment in humans for 15 years). In young adult mice, a 3-month treatment with rapamycin produced a long-lasting effect on the deceleration of intestinal pathology. The effect of rapamycin on lifespan in mice was not measured in this study [1].

In a complementary study, Aiello et al. [2] found that rapamycin treatment immediately after birth increased lifespan in *Mus musculus* (house mouse); the same treatment started later had no effect on lifespan [2]. In mice, the treatment with high doses of rapamycin, from birth to age 30 days, increased median lifespan by 9.6% [2]. The dose was high enough to almost completely block mouse growth. By day 30, rapamycin-treated mice were three times smaller than control mice [2]. Treatment from 30 to 60 days did not increase lifespan [2]. By day 30, control untreated mice reached nearly half the weight of adult mice and that would roughly correspond to the weight of a 10-year-old human.

To literally translate this study to human infants, doses of rapamycin should be high enough to halt growth of the body and the organs. The growth-restricted treatment should last until children are at least 10 years old, an age when normal (untreated) children weigh half that of their adult weight. This is not an option for human longevity.

As shown by Shindyapina et al. [3], rapamycin treatment for 45 days (from the birth to day 45) increased median lifespan of UMHET3 male mice by 11.8% [3]. Rapamycin slowed down mouse growth, decreased organ size (spleen, brain, kidney, liver), and delayed reproduction. Age-related diseases were delayed, and glucose and insulin tolerance tests were improved in the older ages [3]. The study by Shindyapina et al. [3] lacks a lifelong rapamycin-treatment group, so it’s difficult to determine whether transient (45 days) treatment at birth is as effective as lifelong treatment and late-life treatment.

However, based on previous work, we already know that it is not. For example, as shown by Miller et al. [9], started at the age of 9 months, continuous rapamycin treatment (at the same dose of 42 ppm) leads to a 26% increase in median lifespan in females and 23% in males [9] in the same UMHET3 mice as used by Shindyapina et al. [3].

### Middle- and late-life treatment extends lifespan in mice

In numerous studies, rapamycin treatment started at various ages prolonged lifespan and healthspan in mice [9–43].

Rapamycin also extends lifespan when given late in life [10, 11, 34], even transiently for 6 weeks [10] and 3 months [34]. Started at the age of 20 months, continuous everyday treatment with rapamycin prolonged lifespan as efficiently as the identical treatment started at the age of 9 months [11, 14]. Impressively, when started at the age of 20 months, transient (for 90 days) treatment with high-dose rapamycin was sufficient to increase life expectancy by 60% [34]. Started at the age of 2 months, intermittent (every other two weeks) treatment with rapamycin prevented age-related growth, extended lifespan and delayed cancer [12, 13].

### Growth inhibition decelerates aging in growth hormone-deficient mice

**Figure 1:**
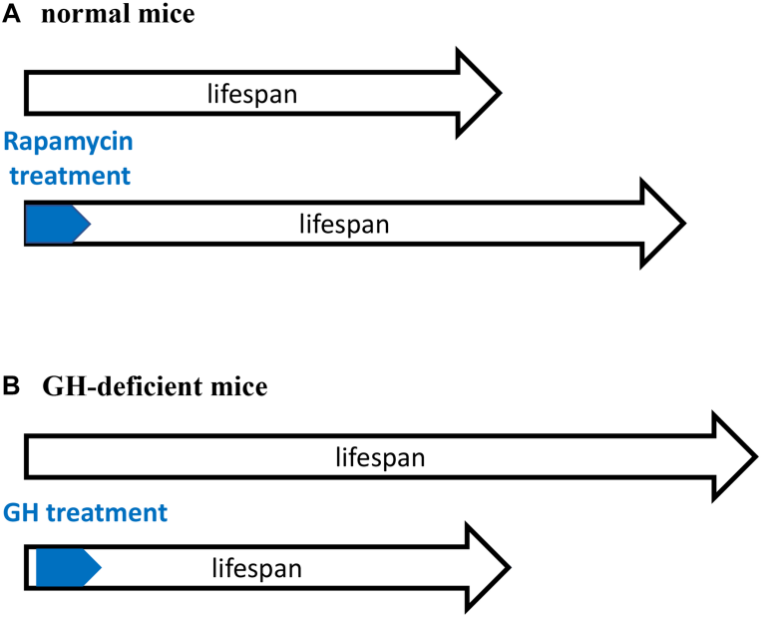
**Critical time window for reprograming of aging by inhibiting developmental growth.** (**A**) Treatment with rapamycin immediately after birth extended lifespan of normal mice. (**B**) Treatment with GH immediately after birth shortened lifespan of GH-deficient mice. GH-deficient mice have low activity of mTORC1. A and B are mirror images showing the same phenomenon.

The existence of an early-life time window that determines the rate of aging [1–3] is supported by previous studies in growth hormone (GH)-deficient mice (Figure 1). GH receptor (GHR) knockout mice and GH-deficient dwarf mice live 40–50% longer than normal mice [44, 45]. Developmental growth of these mice is inhibited, aging is slowed down and the development of age-related diseases is postponed. In contrast, knockout of GHR at 6 weeks of age (when developmental growth is mostly completed) does not increase lifespan [46]. This reveals a “critical developmental time window” (a period of robust growth) that “programs” the rate of aging. In agreement, GH exposure during this period shortens lifespan [47, 48]. Started at age of 2 weeks, administration of GH for a period of 6 weeks accelerated body growth and reverses longevity caused by GH deficiency [47, 49].

Treatment with GH activates mTOR [50]. Importantly the activity of mTOR complex 1 (mTORC1) is low in GH-deficient mice [51–53]. Figuratively, GH/GHR-deficiency is equivalent to rapamycin treatment (Figure 1). Both rapamycin-treated mice and GH-deficient mice have low mTORC1 activity, grow poorly and live longer. Sun et al. suggests that “developmental programming of aging contributes to the developmental origins of adult disease” [47]. The hyperfunction theory suggests the same [4].

### Hyperfunction theory of quasi-programmed aging

According to hyperfunction theory, aging is not caused by accumulation of molecular damage [4–8]. Molecular damage accumulates, of course, but it is not life limiting [54]. It can become life-limiting only when artificially accelerated by knockdown of repair enzymes, for instance [54]. However, in natural organisms, molecular damage is not life-limiting in natural conditions [55]. Instead, life-limiting aging and its diseases are driven by hyperfunctional signaling pathways such as mTOR [4, 5].

The mTOR-driven program is antagonistically-pleotropic; it is beneficial early in life by promoting robust growth at the cost of accelerated aging [56, 57]. mTOR activity is optimal for developmental growth but excessive (hyperfunctional) for longevity. Hyperfunctional signaling pathways such as mTOR and MAPK cause cellular hyperfunctions (e.g., SASP) and systemic hyperfunctions (e.g., hypertension, hyperlipidemia, hyperinsulinemia, hyperglycemia, cellular and organ hypertrophy, hypercoagulation, hyper/autoimmunity, etc), driving age-related diseases. All these result in organ damage and secondary functional decline, a most prominent feature of advanced aging. For example, hyperfunctions such as atherosclerosis, hypertension, hypercoagulation and heart hypertrophy may lead to myocardial infarction and secondary functional decline. Early-stage aging is purely hyperfunctional [4]. Noteworthy, hyperfunction is not necessarily an increase of function. In rare cases, it may be even a decrease, but the function is still higher than needed when growth is completed. In my favorite car analogy, 55 mph on the highway is not hyperfunction but 40 mph on the driveway is. Also, hyperfunctions in some systems coincide with secondary (caused by initial hyperfunctions) functional decline in other systems.

Cellular hyperfunctions drive age-related quasi-programmed diseases that eventually kill any organisms, from worms to humans. [4, 58–68].

### Two approaches to slow aging with rapamycin

**Figure 2:**
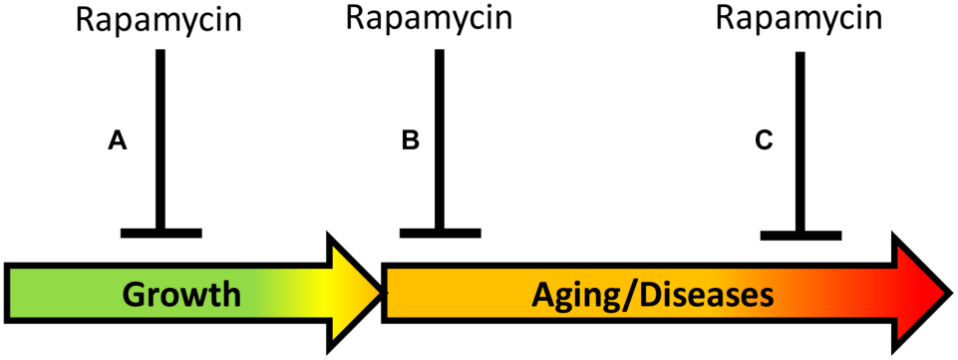
**Timing of rapamycin treatment in some animal studies.** (**A**) Inhibition of growth during development. (**B**) Treatment in the earliest post-development. (**C**) Late-life treatment. Green: growth; Yellow: pre-diseases; Red: age-related diseases.

Despite similar conclusions that transient early-life treatment is sufficient for life extension, three studies present two distinct phenomena and thus, two approaches for life-extension (Figure 2).

Approach A. Aging reprogramming (Figure 2): Inhibiting developmental growth, to slow mTOR-driven aging, which is a continuation of developmental growth. “A critical time window during developmental growth” agrees with the view that aging is quasi-programmed in development [56]. In studies by Aiello et al. [2] and by Shindyapina et al. [3], mice were treated in the phase of rapid growth, immediately after the birth, and rapamycin severely inhibited mouse growth. These studies revealed a “reprogramming window” during developmental growth [2, 3], in agreement with studies in GH-deficient mice [47–49]. Similarly, for life extension, *Daphnia magna* was treated with rapamycin during developmental growth, resulting in a smaller body size [3]. In a study by [2], *Drosophila* was exposed to rapamycin for 3 days during the juvenile growth phase (larva stage till pupal stage), suppressing its growth. The larva was treated before eclosion of the adult fly, and this extended lifespan of the adult fly. A similar approach of treating mice before birth has been suggested [56]. In conclusion, the rate of aging can be decelerated by using rapamycin during the early developmental growth phase. This has important theoretical significance but cannot be implemented in humans.

Approach B (Figure 2): Directly inhibiting mTOR-driven aging in post-development, to postpone age-related diseases and their progression. Aging can be viewed as progression of all age-related quasi-programmed diseases from subclinical to clinical presentation. Direct deceleration of aging in post-development, by inhibiting the same pathways that drive growth in development. In the study by Juricic et al. [1], rapamycin extended lifespan in *Drosophila* treated in early adulthood (for 15 days) but was ineffective late in life, when survival was already decreased. This is consistent with the notion that rapamycin decelerates development of age-related diseases but cannot cure advanced diseases when organs are damaged already. Rapamycin is expected to be most effective to decrease hyperfunction at early stages of diseases, rather than treat terminal stages of diseases associated with loss of function [4]. In fact, early treatment with rapamycin reduced age-related gut pathologies in *Drosophila* and mice later in life [1].

However, numerous studies in mice show that late-life treatment extends lifespan. For example, when started at the age of 20 months, transient (for 90 days) treatment with high-dose rapamycin was sufficient to increase life expectancy by 60% [34]. The difference between *Drosophila* and mice may be explained by different causes of death, since mice do not die from intestinal diseases, mice die from cancer. Also, I suggest that late-life treatment requires higher doses of rapamycin than does early-life treatment. I agree with Matt Kaeberlein that rapamycin was used at suboptimal doses in most studies [69].

### Clinical application

In mice, rapamycin treatment during development severely inhibits growth and decreases the size of organs, including the brain [2, 3]. The treatment is not so brief (30 days [2] and 45 days [3]) in mice. During these 30–45 days, untreated mice reach more than half of their adult weight (humans reach this weight by approximately 10 years old or older). Such an anti-aging treatment would be unacceptable in healthy children because of growth retardation. In children with renal transplant, rapamycin treatment for 12 months significantly decelerated growth [70]. Height growth velocity was decreased from 6.11 cm/year (control) to 4.44 cm/year (rapamycin group) [70].

In humans, anti-aging treatment with rapamycin should be started when growth is completed, to directly inhibit aging, without affecting developmental growth. In post-development, mTOR is hyperfunctional (higher than necessary), a perfect target for inhibition.

On the other hand, studies by Aiello et al. [2], Shindyapina et al. [3] and Juricic et al. [1] emphasize the importance of early-life treatment. One may suggest that treatment with rapamycin should not be unnecessarily delayed if we want to extend lifespan reliably.

**Figure 3:**
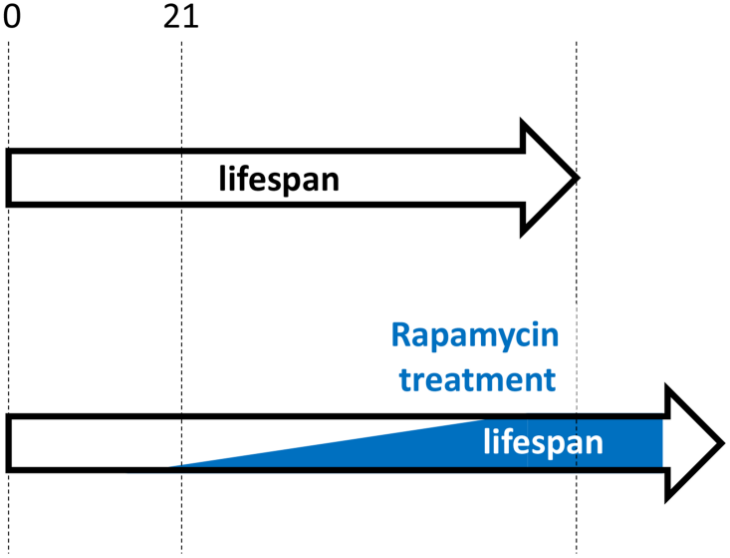
**Hypothetical rapamycin treatment in humans for maximal longevity.** Started early in post-development (for example, at 21 yo), low doses of rapamycin decelerate progression of pre-diseases (slow aging). Side effects are more undesirable at younger ages and doses should be low. Doses are gradually increased, to avoid rapamycin adaptation and to maximize therapeutic potential.

Similarly, as suggested in 2006, “As an anti-aging drug, rapamycin prevents age-related diseases rather than cure complications of diseases. Rapamycin will prevent organ failure but not reverse it. … rapamycin will be most useful to slow down senescence and to prevent diseases” [4]. Treatment should be started early in life but not earlier than growth is completed (Figure 3). For example, rapamycin may be considered from the age of 21–25 (just an example). This may seem at odds with the work showing rapamycin treatment started at the age of 20 months (old mice) was as effective as treatment started at the age of 9 months [11, 14]. However, this result should not be overgeneralized, as the result may depend on specific conditions, mouse strains and doses. In fact, this was challenged by additional experiments (rapamycin plus acarbose) by the same authors [43]. Also, adaptation to rapamycin may explain the result. I suggest that an early-onset treatment in post-development with low doses that would be gradually increased to maximal anti-aging doses by the age of 50 (an arbitrary age) would be most effective. Whether rapamycin should be taken at high intermittent doses (for example, 12 mg every other week) or chronically (for example, 0.5–1 mg every day) will be discussed in my forthcoming paper. Both regimes have cons and pros, but not those that are commonly assumed.

### Disclaimer

This article is intended for a professional audience. This article does not represent medical advice or recommendations to patients.
